# The Incidence of Amikacin Ototoxicity in Multidrug-ResistantTuberculosis Patients

**Published:** 2011

**Authors:** Mohammad Reza Javadi, Bahareh Abtahi, Kheirollah Gholami, Behzad Safari Moghadam, Payam Tabarsi, Jamshid Salamzadeh

**Affiliations:** a *Department of Clinical Pharmacy, School of Pharmacy,Tehran University of Medical Sciences, Tehran, Iran.*; b *School of Pharmacy, Shahid Beheshti University of Medical Sciences, Tehran, Iran.*; c *National Research Institute of Tuberculosis and Lung Diseases, Masih Daneshvari Hospital, Shahid Beheshti University of Medical Sciences, Tehran, Iran.*

**Keywords:** Ototoxicity, Amikacin, Multidrug-resistant tuberculosis, Pure-tone audiometry

## Abstract

Amikacin has been shown to irreversibly suppressCochlear activity.The aim of this study is to assess the incidence of amikacinototoxicity in multidrug-resistant tuberculosis patients and riskfactors associated withthis ototoxicity.In this cross-sectional study, 41 patientswith multidrug-resistant tuberculosis (MDR-TB) were included.All patients received fixed dose of intravenous amikacin(500 mg/day) and anti-TB medications for six months. Baseline Pure-Tone Audiometry (PTA) was performed on all patients,before and during the drug treatment with the frequency range between 250 Hz and 8000 Hz. Patients were closely observed for the occurrence of symptomatic ototoxicity using a questionnaire .To find an association between the incidence of cochlear damage and patients’ demographics, all patients’ data were recorded.

A total of 29 patients suffered from hearing loss (70.1%) (Male: n = 18; Female: n = 20).Using logistic regression, the incidence ofamikacinototoxicity was higher in men than in women. There was a negative correlation between the duration of the amikacin treatment and the difference in hearing thresholds(r = -0.34, p = 0.03). The mean of hearing threshold was significantly increased before and after the amikacin treatment((23.68 ± 19.26 vs. 38.93 ± 22.80) (p < 0.0001)). The incidence of hearing loss was remarkable in MDR-TB patients treating with amikacin. However, risk factors’ determination and monitoring of audiometric result variations could haveinfluenced the incidence of the amikacin ototoxicity.

## Introduction

Amikacin, a semi-synthetic aminoglycoside (AG), shows considerable activity againstmultidrug-resistant tuberculosis (MDR-TB) compared to the other AGs, with a similar ototoxicity to that of gentamicin (1-4). Aminoglycoside ototoxicity in both human and experimental models was defined as damage inthe auditory system, vestibular systemor both (5-7). Among AGs, streptomycinandgentamicinhave higher rates of vestibulotoxicity (gentamicin has both vestibulotoxicity and cochleotoxicity but its’ vestibulotoxicity is more pronounced), while amikacin, neomycin, dihydrostreptomycin, and kanamycin have higher rates of cochleotoxicity. Cochleotoxicity is generally a more serious problem than the vestibulotoxicity which leads to a permanent sensorineural hearingloss (8, 9). Studies showed thatthis toxicity affects audiometrically detectable high-frequency hearing earlier than low-frequency oneswhich encompass the speech (10-13).

MDR-TB was defined as a high-level resistance to both isoniazidand rifampin, with or without resistance to other anti-TBdrugs (14). Chemotherapy of MDR-TB is based on the administration of first-line oral drugs combined with the additional injectable aminoglycosides (AGs), fluoroquinolones, oral bacteriostatic second-line drugs, and anti tuberculosisagents with unclear efficacy (15). However, treatment usually involves potentially toxic drugs andas a result, it is controversial whetherthe MDR-TB is treatable or not (16-21).

Different factors, such as lack of adequate audiological testing technology and lack of standards for hearing loss, which consider differentauditory thresholds for ototoxicity, emerge a need forfurther investigations (10). To our knowledge, this is one of the first studies that investigatesthe relationship between patients’ characteristics and the incidence of the amikacinototoxicity in MDR-TBthus,there is a little data related to the incidence of the ototoxicity of amikacin in this group ofpatients.The aim of this study was to investigate the incidence of ototoxicity in MDR-TB patients and study the risk factors that would influence thistoxicity.

## Experimental

All 41 patients receiveda fixed dose of intravenous amikacin(500 mg daily as 30 min IV infusion over 30 min) combined with the second- and third-line drugs for MDR-TBthrough six months. Those patients with any pre-treatment evidence of hearingloss (congenital deafness) and congenital abnormalities and also patients with evidence of infective pathology in ear (meningitis,chronic otitis media), surgical procedures and the ones using concomitant ototoxic drugs were excluded from our study.

Baseline Pure-Tone Audiometry (PTA) was performed on all patients before and during the drug treatment. Baseline pure-tone audiograms between 250 Hz and 8000 Hz were performed for all the patients in anacoustic room.They were observed closely for the occurrence of symptomatic ototoxicity including deafness,tinnitus and dizziness using questionnaires and consultations with the medical specialist. All information regarding to the patients’ profile,the relation between amikacin and the incidence ofototoxicity, the way of countering with toxicity (*e.g.*drug discontinuing)and the severity of toxicity, wererecorded.

Ototoxicity is damage to the ear, specifically the cochlea or auditory nerve and sometimes the vestibular system.The criteria used for determining ototoxicity threshold shifted from baseline audiogram were: (I) 20 dB or greater decrease at any one test frequency, (II) 10 dB or greater decrease at any two adjacent frequencies, or (III) loss of response at three consecutivefrequencies.

To investigate the influence ofdemographics, renal and liver functions and concomitant medications on amikacinototoxicity, all the patients’ data were recorded on MDR-TB patient sheets at the baseline and during the treatment.

Continuous data were tested for normal distribution by the Kolmogorov-Smirnov test. An independent sample t-test was used for data in normal distribution and the Mann-Whitney U-test was used for data in abnormal distribution. Continues data are presented as mean ± SD and range, according to variable distribution. Logistic regression was performed to investigate the probability that a person has a ototoxicity within a specified time period predicted from knowing of the person›s age, sex, body mass index, cigarette smoking, nationality andhistory of ototoxicity (baseline hearing threshold > 25 dB),renal and liver functions.Analysis of the relation between the variables with increase in hearing threshold was performed using multiple regressions. Statistical analysis of data was performed by χ^2^ test (chi-square test) and the Fisher exact probability test (2-tailed); p-valuesless than 0.2 were considered statically significant. Statistical analysis was performed using SPSS software for windows (version 10 USA).

## Results and Discussion

A total of 41 patients were enrolled in our study (Male: n = 20; Female: n = 21) with age group between 15 to 74 years (38.29 ± 17.05), who had confirmed MDR-TB from July 2003 to June 2004 (45.07 ± 27.67 days) in Masih Daneshvari Hospital located in Iran. The mean time for the investigation of ototoxicity incidence was 3 months.Twenty-nine of 41amikacin recipients (70.1%) experienced decreases of at least 20dB on at least one occasion; 10dB or greater decrease at any two adjacent frequencies, or loss of response at three consecutive frequencies. There was no difference in the mean daily dose administered to inpatient populations with or without toxicity (500 mg/day). Demographics of whole patients and also the ones with ototoxicity, according to the possible potentially contributing factors, are shown in Table1.

**Table 1:** Patients’ characteristics

**Characteristics**	**Whole patients (n = 41)**	**Patients with ototoxicity (n = 29)**
Age (years)	(15-74) 38.29 ± 17.05	(15-74) 41.13 ± 18.13
Sex (F/M)	21/20	11/18
Weight (Kg)	(34-80) 49.13 ± 10.10	(34-80) 49.45 ± 11.54
BMI (Kg/m²)	(12-34) 19.2 ± 4.06	(12-34) 18.95 ± 5.02
Nationality (Afghani/Iranian)	20/21	17/12
Duration of treatment with amikacin (days)	(13-131) 45.07 ± 27.67	(13-102) 35.97 ± 19.55
Cigarette smoking	9	8
Drug abuse	7	6
Alcohol consumption	3	3
History of the pervious ototoxicity	19	17
Concomitant medications (in/de)	32/9	23/6
Renal impairment	1	1
Liver impairment	9	7

F: Female; M: Male; Concomitant medications, in: Increase the amikacin blood concentrations (*e.g. *NSAIDS); Concomitant medications, de: Decrease the amikacin blood concentrations (*e.g*. Penicillins); Data about the history of ototoxicity was obtained by patients’ interview at the beginning of study

Thehearing impairment was bilateral in 18 patients (62.06%) and unilateral in 11 patients (n = 6, right ear and n = 5, left ear). The severity of ototoxicityvaried widely from patient to patient (mild ototoxicity: about 44.83%; moderate ototoxicity: 17.24%; moderately severe ototoxicity: 24.14%; severe ototoxicity: 10.34%; profound ototoxicity: 3.45% .

To investigate the relationship betweenthe individual demographics and the incidence of ototoxicity, patients were divided in two groups: those with ototoxicity (n = 29) and those without ototoxicity (n = 12).The effect of Weight, BMI, concomitant medications, cigarette smoking and alcohol use were not significant between two groups (p > 0.2),while there was a significant difference in terms of sex, nationality and the pervious history of ototoxicity between patients with auditory toxicity and those with no change in audiograms (p < 0.2) (Table 2). 

**Table 2:** The relationship between the risk factors and the incidence of ototoxicity

**Factors**	**Test**	**p-value**
Age (years)	Mann-Whitney	0.2
Sex (F/M)	Chi-square	0.008
Weight (Kg)	Mann-Whitney	0.96
BMI (Kg/m²)	Mann-Whitney	1
Nationality (Afghanian/Iranian)	Chi-square	0.14
History of Ototoxicity	Chi-square	0.014
Concomitant medications (in/de)	Fisher´s Exact test	1
Drug abuse	Fisher ´s Exact test	0.65
Cigarette smoking	Fisher´s Exact test	0.24
Renal impairment	Chi-square	0.85
Liver impairment	Fisher´s Exact test	0.45

F: Female; M: Male; BMI: Body Mass Index; in: Increase the amikacin blood concentrations (*e.g. *NSAIDS); de: Decrease the amikacin blood concentrations (*e.g*. Penicillins

After investigating the interactions of these factors (age, sex, nationality and the pervious history of ototoxicity) (Table3), logisticregression test was performed. According to the final model, the only factor that showed a significant association with the development of ototoxicity was sex where men were more prone to ototoxicity than women (90% vs. 52.38%) (Equation 1).

*(X = 1, men and X = 2, women)*

$\frac{\mathrm{pr}\mathrm{ob}.\mathrm{ototoxicity}}{\mathrm{pr}\mathrm{ob}.\mathrm{No}\mathrm{ototoxicity}}=0.095\times e^{-2.1}x_{1}$

**Table 3:** The relationship between the risk factors and the incidence of ototoxicity

**Factors**	**Test**	**p-value**
**Age Χ Sex**	Mann-Whitney	NS
**Age Χ Nationality**	Mann-Whitney	0.003
**Age Χ History of Ototoxicity **	Mann-Whitney	NS
**Nationality Χ Sex **	Chi-square	NS
**History of Ototoxicity Χ Sex **	Chi-square	0.02
**History of Ototoxicity Χ Nationality**	Chi-square	NS

NS: Not Significant

The hearing thresholds before and after the drug treatment were not different significantly in association with age, weight, BMI, history of ototoxicity, concomitant medications, drug abuse or cigarette smoking (p > 0.2), while hearing threshold was influenced by sex and the length of amikacintreatment and also the factor of having Afghani nationality (p < 0.2) (Table 4). Using multiple regression, there was a linear relationship between the duration of amikacintreatment and the difference in hearing thresholds (r = -0.34, p = 0.03); the meanofhearing thresholdwassignificantlyincreasedafter theamikacin treatment (23.68 ± 19.26 *vs. *38.93 ± 22.80, p < 0.0001).

**Table 4:** The relationship between the risk factors and the increase of hearing threshold

**Factors**	**Test**	**p-value**
**Age (years)**	Kendall´s Rank correlation	0.37
**Sex (F/M)**	Mann-Whitney	0.16
**Weight (Kg)**	Kendall´s Rank correlation	0.91
**BMI (Kg/m²)**	Kendall’s Rank correlation	0.851
**Nationality (Afghanian/Iranian)**	Mann-Whitney	0.76
**History of ototoxicity**	Mann-Whitney	0.34
**Concomitant medications (in/de)**	Mann-Whitney	0.6
**Drug abuse**	Mann-whitney	0.8
**Cigarette smoking**	Mann-whitney	0.55
**Duration of treatment**	Kendall’s Rank correlation	0.13

F: Female; M: Male; BMI: Body Mass Index; in: Increase the amikacin blood concentrations (*e.g. *NSAIDS); de: Decrease the amikacin blood concentrations (*e.g*. Penicillins).

In this study, age, cigarette smoking or drug abuse, do not significantly associate with the incidence and progress of cochleotoxicity, while similar to Black *et al. *study, our patients who were previously treated with amikacin, were more likely to develop hearing loss (20).

The Naranjo adverse drug reaction (ADR) probability scale was used and a score of 7 was obtained, indicating a probable ADR from amikacin. There were significant advances in understanding the mechanisms underlying the aminoglycoside ototoxicity in the past decade. It is now possible to identify the individuals with a genetic susceptibility to aminoglycoside ototoxicity, which can prevent a significant proportion of cases with hearing loss after aminoglycoside exposure. The knowledge resulting from studies has also expanded understanding the role of reactive oxygen species in a broad range of inner ear pathologies. In practical terms, knowledge of the mechanism will drive the design of novel rational therapeutic interventions. The amelioration of adverse effects of aminoglycosides will have far reaching implications for the safe use of drugs whose primary efficacy is unquestioned.

The cochleotoxicityof AGsis not easily detectable and is mostly not with any clinical symptoms; while there isno recommendation for therapeutic drug monitoring in MDR-TB patients using amikacin. In this study, 29 patients among 41 (70.1%) experienced ototoxicity detected by 20dB or greater decrease at any one test frequency, 10dB or greater decrease at any two adjacent frequencies, or loss of response at three consecutive frequencies using serial audiograms. This decrease in standard PTA had an interindividual variability between 6 and 50dB, compared to Duggal *et al. *study with similar hearing losscriteria in MDR-TB patients receiving second line AGs (Amikacin, capreomycin, kanamycin) where 18.7% of patients developed sensorineural hearing loss (15). De Jager *et al. *study showed hearing loss in 13 MDR-TB patients (21.3%) out of 70 using streptomycin, amikacin or kanamycin with decrease in 20dB at any one test frequency or 15dB at two or more consecutive frequencies (23). Studies in cystic fibrosis patients showed that the incidence of AGototoxicitywas 17% and 24%, respectivelywith a different hearing loss standard criteria: decrease in 15dB to 20dB at two or more consecutive frequencies in comparison with thedecrease in 15dB any one test frequency (22, 24). The incidence of ototoxicity in patients with Gram-negative infections treated with amikacinwas 28.5%, however the decrease in 15dB at any one test frequency considered as drug toxicity (25). The lack of acceptable standards to determine the hearing loss leads to differences in the result of our study,in comparison with other studies. Symptomatic ototoxicity including deafness, tinnitus and dizziness did not occur in our patients and most of them were not aware of their hearing loss, even when this decrease reached to lower frequencies.

Our results showed that the difference of hearing threshold, before and after the treatment, was negatively correlated with the duration of amikacintreatment.The severity of the hearing loss was decreased by the time. Interestingly, this finding was a contrast to other studies which reported thatby an increaseinamikacin administration days, the severity of hearing loss had increased (22, 24). Our finding could be explained bythe fact that cochleotoxicityleads to a destruction of cochlear hair cells by generating oxygen and nitrogen free-radical species, which initiate an apoptosisintrinsic pathway cascade in hair cells (26-28). However,one study in an animal model, showed that following chronic AG ototoxicity regulatory systems including high expression of Fas protein wouldcontrol the hair cells apoptosis. In addition,through increasing the duration of treatment,there will be an increase inthe uptake of drug by cochlear outer and innerhair cells (OHCs, IHCs) and also a biphasic release from these cells, thus, free radicals generated throughcochleotoxicity would be removed bydetoxicant systems and the repeated AG exposure could cause the “upregulation” of detoxicant systems in the OHCs and/or IHCs (24).

Different studies investigated risk factors including largetotal daily dose, repeated courses of treatment, length of treatment, pervious ototoxicity history, renal function, age, sex, extracellular fluid volume,hematocrit and body mass index contributing to ototoxicity of AGs (22, 24, 25, 30-34). To our knowledge, this is one of the first studies that evaluated the influence of these factors in MDR-TB patients receiving AG.In addition, our study–like thestudy of Braza*et et al. *showed the higher incidence of ototoxicity in men in comparison withwomen (the incidence of hearing loss in men was about 8.2 times more than women) (25).One reason for our finding could be the low rate of elimination and longerhalflife of AGs in men comparing to women (34, 35).

Our patients received amikacin only for a course andas a result, we could not find a relationship between amikacin ototoxicity and repeated courses of treatment. In addition, total daily dose of amikacin was not large and was similar in patients with or without toxicity (500 mg/day), which could not be considered as a variable. Since only one patient in ototoxic groupexperienced renal failure during the therapy,we could not conclude whetherthe renal disease is a predisposing factor for ototoxicity. A disadvantage of these studies was that we did not obtain amikacin serum levels and serum creatinine for any of our patients. So, interindividualdifferences in amikacin clearance were not evaluated.

In conclusion, as the patients generally had no sign of ototoxicity and were unaware of any hearing loss, they could not be expected to report their hearing loss. The lack of a reliable audiological testing and hearing loss standard determinants, emphasizes the need to observe precautions in patients using amikacin. Our data suggest that it is essential to conduct furtherstudies ina larger population of MDR-TB patients who received amikacin to investigate different variables contributed to ototoxicity. 
